# Current Trends in Targeted Therapies for Glioblastoma Multiforme

**DOI:** 10.1155/2012/878425

**Published:** 2012-03-05

**Authors:** Fumiharu Ohka, Atsushi Natsume, Toshihiko Wakabayashi

**Affiliations:** Department of Neurosurgery, Nagoya University School of Medicine, 65 Tsurumai-cho, Showa-ku, Nagoya 466-8550, Japan

## Abstract

Glioblastoma multiforme (GBM) is one of the most frequently occurring tumors in the central nervous system and the most malignant tumor among gliomas. Despite aggressive treatment including surgery, adjuvant TMZ-based chemotherapy, and radiotherapy, GBM still has a dismal prognosis: the median survival is 14.6 months from diagnosis. To date, many studies report several determinants of resistance to this aggressive therapy: (1) *O* ^6^-methylguanine-DNA methyltransferase (MGMT), (2) the complexity of several altered signaling pathways in GBM, (3) the existence of glioma stem-like cells (GSCs), and (4) the blood-brain barrier. Many studies aim to overcome these determinants of resistance to conventional therapy by using various approaches to improve the dismal prognosis of GBM such as modifying TMZ administration and combining TMZ with other agents, developing novel molecular-targeting agents, and novel strategies targeting GSCs. In this paper, we review up-to-date clinical trials of GBM treatments in order to overcome these 4 hurdles and to aim at more therapeutical effect than conventional therapies that are ongoing or are about to launch in clinical settings and discuss future perspectives.

## 1. Introduction

Glioblastoma multiforme (GBM) is one of the most frequently occurring tumors in the central nervous system and the most malignant tumor among gliomas. A subanalysis in an international randomized trial by the European Organization for Research and Treatment of Cancer/National Cancer Institute of Canada (EORTC/NCIC) compared the results of radiotherapy (RT) alone with those of concomitant RT and temozolomide (TMZ) and found that the addition of TMZ to radiotherapy for newly diagnosed GBM resulted significant survival benefit [1], additionally the subgroup analysis of the 5-year survival data of the EORTC/NCIC trial also revealed its benefit [2]. Since then, TMZ has been the current first-line chemotherapeutic agent for GBM. However, despite aggressive treatment including surgery, adjuvant TMZ-based chemotherapy, and RT, GBM still has a dismal prognosis: the median survival is 14.6 months from diagnosis. Many studies aim to overcome several determinants of resistance to conventional therapy by using various approaches to improve the dismal prognosis of GBM such as modifying TMZ administration and combining TMZ with other agents, developing novel molecular-targeting agents, and novel strategies targeting GSCs. In this paper, we review up-to-date clinical trials of GBM treatments in order to overcome determinants and to aim at more therapeutical effect than conventional therapy that are ongoing or are about to launch in clinical settings and discuss future perspectives.

## 2. Overcoming Alkylating Agent Resistance due to MGMT

MGMT is capable of counteracting the cytotoxicity induced by *O* ^6^-alkylating agents. Furthermore, increased MGMT expression is well correlated with *in vitro* and *in vivo* glioma resistance to TMZ [3–6]. However, in this process, MGMT is rapidly degraded via the ubiquitin-proteasome pathway after receiving alkyl groups from DNA; the repletion of cellular MGMT pools also depends on the resynthesis of the molecule [7]. This makes MGMT a suitable target for intervention to improve the therapeutic efficacy of TMZ.

Additional treatment options are limited in cases of relapse after a standard-dose TMZ treatment (150–200 mg/m^2^ × 5 days, q4weeks). Supported by the assumption that continuous treatment with alkylating agents induces the depletion and exhaustion of MGMT activity, many researchers have investigated the effects of different dose- and time-modified TMZ schedules.

### 2.1. RTOG 0525/EORTC 26052-22053 (Dose-Dense) Study

**Figure 1:**
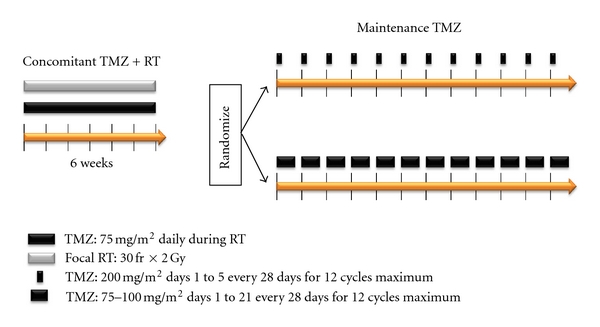
RTOG 0525/EORTC 26052-22053 (dose-dense) study. The study aimed to determine if intensified TMZ (75–100 mg/m^2^  × 21 days, q4weeks) improves overall survival or progression-free survival compared to the standard arm (150–200 mg/m^2^  × 5 days, q4weeks).

This is a randomized phase III trial comparing standard adjuvant TMZ with a dose-dense schedule in newly diagnosed GBM [8]. This trial was based on a report indicating that dose-dense TMZ prolongs MGMT depletion in blood mononuclear cells and possibly tumors; the study aimed to determine if intensified TMZ (75–100 mg/m^2^ × 21 days, q4weeks) improves overall survival (OS) or progression-free survival (PFS) compared to the standard arm (150–200 mg/m^2^ × 5 days, 4weeks) after the standard concomitant RT+TMZ (Figure 1). No significant difference was observed between the standard and experimental arms with respect to median OS (16.6 versus 14.9 months, *P* = 0.63), median PFS (5.5 versus 6.7 months, *P* = 0.06), or *MGMT* methylation status. In addition, the experimental arm significantly increased grade ≥3 toxicity including lymphopenia and fatigue. This study did not demonstrate improved efficacy of dose-dense TMZ for newly diagnosed GBM regardless of *MGMT* methylation.

### 2.2. Continuous Dose-Intense TMZ in Recurrent Malignant Glioma: The RESCUE Study

**Figure 2:**
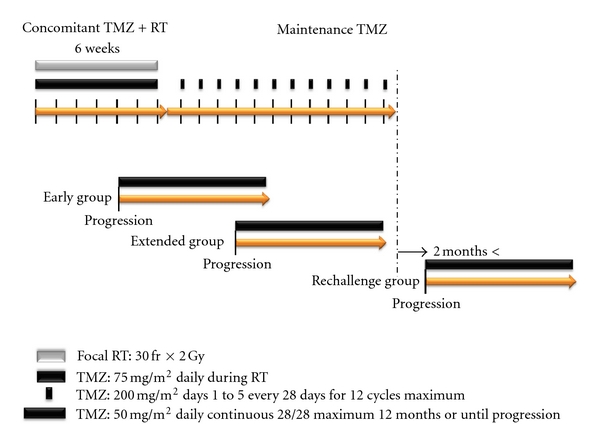
Continuous dose-intense TMZ in recurrent malignant glioma: the RESCUE study. Ninety-one patients with GBM were prospectively divided into 3 groups according to the timing of progression during adjuvant therapy: early, extended, and rechallenge.

There is no consensus on the optimal approach for patients with recurrent GBM, in which recurrence occurs after TMZ is initially used followed by 12 or more cycles of adjuvant therapy. Protracted drug exposure may reduce MGMT activity as described above. In addition, protracted TMZ dosing may inhibit endothelial cell recovery in the tumor and the activity of circulating endothelial precursors as well as upregulate thrombospondin-1, leading to an antiangiogenic effect [9–12]. Ninety-one patients with GBM were prospectively divided into 3 groups according to the timing of progression during adjuvant therapy: early, extended, and rechallenge [13] (Figure 2). All patients received 50 mg/m^2^/day TMZ on a continuous (28/28) basis for a maximum of 12 months or until progression occurred. The primary endpoint of this study was 6 months PFS (PFS6). PFS6 was 27.3%, 7.4%, and 35.7% in the early, extended, and rechallenge groups, respectively; 1-year survival from time of study entry was 27.3%, 14.8%, and 28.6% for the 3 groups, respectively. The results of the RESCUE study suggest that patients who progress early compared with those who progress late or after a treatment-free interval may respond differently to the continuous dose-intense TMZ re-treatment. However, given that no consensus treatment option exists for patients with recurrent GBM, it would be of note that continuous dose-intense TMZ serves as a useful platform for combination strategies.

### 2.3. One-Week-on/One-Week-off TMZ in Elderly Patients with Newly Diagnosed Malignant Gliomas: The NOA-08 Study

Both surgery and radiation therapy are less tolerated in elderly patients than in younger ones. To reevaluate the widespread therapeutic nihilism with malignant glioma in the elderly (age > 65), the Neurooncology Working Group (NOA) of the German Cancer Society conducted a randomized phase III trial to compare a 1-week-on/1-week-off TMZ schedule at 100 mg/m² with dose modification in 25 mg steps in both directions and involved field RT (54–60 Gy) in elderly patients with newly diagnosed anaplastic astrocytoma or GBM (NOA-08) [14]. The primary endpoint was the median OS during follow-up in the 12 months after the operation. Patient characteristics were balanced between arms in the intention-to-treat population (*n* = 373) except for more resections and more anaplastic astrocytomas in the RT arm. TMZ was not demonstrated to be superior. However, patients in the TMZ arm had an increased risk of death (HR = 1.24 [95% CI: 0.94–1.63]) compared with those in the RT arm. The rates of adverse and serious adverse events were also higher in the TMZ arm. This trial failed to demonstrate the noninferiority of dose-intensified TMZ alone compared with RT alone in the primary treatment of older patients with malignant gliomas. Whether RT plus TMZ is superior to RT alone, is being addressed in an ongoing companion trial conducted by the NCIC, EORTC, and RTOG.

### 2.4. Combination with 1,3-Bis (2-Chloroethyl)-1-Nitrosourea: The TEMOBIC Study

Although TMZ replaced nitrosoureas such as 1,3-*bis* (2-chloroethyl)-1-nitrosourea (BCNU) as the standard initial chemotherapy in the treatment of GBM, the DNA damage induced by nitrosoureas and TMZ is partially repaired by MGMT. Thus, combined administration of nitrosoureas and TMZ might overcome MGMT-mediated resistance via MGMT depletion, yielding superior treatment results compared with the administration of TMZ alone. A phase II study was conducted in newly diagnosed anaplastic oligodendrogliomas [15]. This study assessed the efficacy and safety of BCNU (150 mg/m^2^, day 1) and TMZ (110 mg/m^2^, days 1–5) every 6 weeks for up to 6 cycles before conventional RT (60 Gy/30 fr) in 54 patients. Grade 3-4 toxicities included thrombopenia (*n* = 20), neutropenia (*n* = 13), and elevated transaminases (*n* = 5). Treatment was discontinued in 4 patients, and possible treatment-related deaths occurred in 3 patients. The combination should be carefully considered due to its significant toxicity.

### 2.5. Combination with *O* ^6^-Benzylguanine

*O* ^6^-benzylguanine (*O* ^6^-BG) is a potent inhibitor that irreversibly inactivates MGMT by covalently transferring its benzyl group to the cysteine residues of MGMT's active site [16]. As a result, *O* ^6^-BG enhances TMZ cytotoxicity in MGMT-proficient glioma cells both *in vitro* and *in vivo* but not in MGMT-deficient cells [17]. Since patients with MGMT overexpression in tumors respond more poorly to alkylating agents, co-administration of *O* ^6^-BG to deplete the tumor pools of MGMT to enhance drug cytotoxicity has been previously attempted in a clinical setting [18, 19]. However, systemic delivery of *O* ^6^-BG increased the myelotoxicity caused by MGMT depletion in bone marrow cells. Therefore, the dose of alkylating agents was reduced to a subtherapeutic level. Consequently, none of the patients responded to this drug combination. Therefore, the therapeutic potential of adding *O* ^6^-BG to enhance TMZ cytotoxicity in tumor cells has been discouraging thus far.

### 2.6. Combination with Cilengitide: The CORE Study

Cilengitide, a selective *α*v*β*3/5 integrin inhibitor, exhibits dose-dependent antitumor activity in patients with recurrent GBM [20]. A randomized controlled phase II trial (CORE) was designed as stepwise cilengitide intensification in subjects with newly diagnosed GBM and unmethylated *MGMT* promoter status [21]. The toxicity from this treatment was minimal. A further phase trial testing the use of intensified cilengitide (2000 mg, 5 days/week) in combination with concomitant RT with TMZ is now recruiting patients with newly diagnosed GBM with unmethylated *MGMT* status.

### 2.7. Combination with Interferon-*β*: The JCOG0911 (INTEGRA) Study

Interferon (IFN)-*β* exerts pleiotropic biological effects and is widely used either individually or in combination with other antitumor agents to treat malignant gliomas and melanomas [22]. In the treatment of malignant gliomas, IFN-*β* can act as a drug sensitizer by enhancing the toxicity of chemotherapeutic agents against various neoplasms when administered in combination with nitrosoureas. Combination therapy with IFN-*β* and nitrosoureas is primarily used for the treatment of gliomas in Japan [23]. In a previous *in vitro* study in human glioma cells, we found that IFN-*β* markedly enhances chemosensitivity to TMZ [24]; this finding suggests that one of the major mechanisms by which IFN-*β* enhances chemosensitivity is the downregulation of MGMT transcription via p53 induction. This effect was also observed in an experimental animal model [25]. The results of these 2 studies suggest that compared with chemotherapy with TMZ alone and concomitant RT, chemotherapy with IFN-*β* and TMZ with concomitant RT might further improve the clinical outcomes of malignant gliomas. To evaluate the safety, feasibility, and clinical effectiveness of combination therapy with IFN-*β* and TMZ, a phase I clinical study, the Integrated Japanese Multicenter Clinical Trial: a Phase I Study of Interferon-*β* and TMZ for Glioma in Combination with RT (INTEGRA study), was conducted. The primary endpoint was the incidence of adverse events. The exploratory endpoints were PFS and OS. The study population comprised 16 patients with newly diagnosed gliomas and 7 with recurrent high-grade gliomas. Grade 3-4 leukocytopenia and neutropenia were observed in 6.7% and 13.3% of the patients, respectively. Overall, 40% of the patients exhibited an objective response to therapy. In patients with newly diagnosed GBM, the median OS time was 17.1 months and the rate of 1-year PFS was 50%.

**Figure 3:**
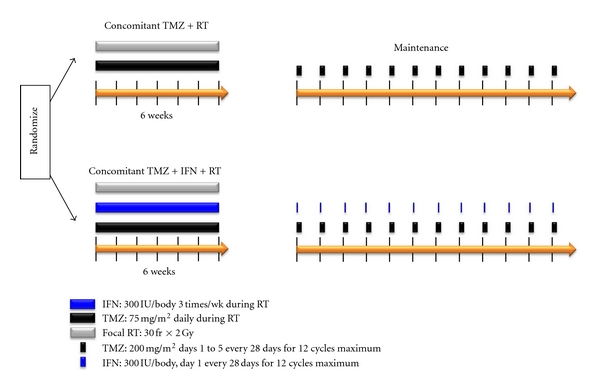
Combination with interferon-*β*: the JCOG0911 (INTEGRA) study. A randomized phase II clinical trial in patients with newly diagnosed GBM is under way to compare the standard-of-care regimen with the addition of IFN-*β* in the upfront settings.

This regimen is safe and well tolerated by the patients, and may prolong the survival of patients with GBM. A randomized phase II clinical trial in patients with newly diagnosed GBM is under way to compare the standard-of-care regimen with the addition of IFN-*β* in the upfront settings (Figure 3).

## 3. Strategies Targeting the Altered Signaling Pathways (Figure 4)

### 3.1. VEGF Signaling

GBM is characterized by sustained angiogenesis—the key regulator of which is vascular endothelial growth factor (VEGF). Bevacizumab (Avastin, BEV) is a humanized monoclonal antibody that binds to and inhibits the activity of VEGF [26–29]. In preclinical models, BEV has been shown to exhibit activity against GBM both alone and in combination with RT and TMZ. The benefit and safety profile of BEV was confirmed in a randomized, noncomparative phase II trial (BRAIN study; AVF3708g) in GBM patients who experienced first or second recurrence following standard-of-care with TMZ [27]. In both the BEV (*n* = 85) and BEV plus irinotecan (*n* = 82) cohorts, objective response rate and PFS6 were significantly higher than those of the external historical controls. It might be important to state that the addition of irinotecan to bevacizumab did not improve outcome, and PFS6 as a primary endpoint is a controversial particularly in patients treated with antiangiogenic therapies (such as bevacizumab) that can lead to improved imaging findings without actual tumor response (a so-called pseudoresponse). In any case, in light of these results, studies were initiated to evaluate BEV in combination with RT plus TMZ as the upfront treatment for newly diagnosed GBM. In a noncomparative study in 70 newly diagnosed GBM patients, BEV in combination with RT plus TMZ resulted in median OS and PFS times of 19.6 and 13.6 months, respectively. In another nonrandomized phase II study [30], the effects of RT plus TMZ were compared with (*n* = 25) and without (*n* = 31) BEV; RT plus TMZ with BEV resulted in increased PFS6 (87% versus 52%), median PFS (12 versus 7 months, *P* = 0.0001), 2-year OS rate (50% versus 22%), and median OS (24.0 versus 17.5 months, *P* = 0.09). A large randomized double-blind placebo-controlled phase III trial (AVAglio, BO21990, NCT00943826) is currently recruiting approximately 920 newly diagnosed GBM patients from 140 centers worldwide [31]. However, there is evidence suggesting that anti-VEGF treatment increases tumor cell invasion in GBM [32]. While BEV strongly decreases contrast enhancement, this is accompanied by a 68% increase in infiltrating tumor cells in the brain parenchyma. These data strongly suggest that vascular remodeling induced by anti-VEGF treatment leads to a more hypoxic tumor microenvironment. This favors a metabolic change in the tumor cells toward glycolysis, which leads to tumor cell invasion into normal brain tissue.

Cediranib is another potent oral VEGF signaling inhibitor that exhibits activity against all 3 VEGF receptors [33]. REGAL (NCT00777153) is a randomized phase III study comparing cediranib and lomustine (CCNU) in patients with recurrent GBM. Between October 2008 and September 2009, 325 patients from 10 countries were enrolled. The first clinical results of the REGAL trial were negative [34]. Other clinical trials are under way to assess cediranib either as a monotherapy or in combination with other agents.

### 3.2. EGFR and PDGF Signaling

To date, various genetic alterations are reported in GBMs such as e*pidermal growth factor receptor* (*EGFR*) amplification, *CDKN2A *loss, *phosphatase and tensin homolog* (*PTEN*) loss, and so forth. Among these various alterations, several alterations deregulate pathways involving the RTK/PI3K/Akt/mTOR pathway [36, 37], which is regarded as the most amenable pathway to pharmacologic intervention [38]. This pathway contains a number of key kinase intermediates as shown below. *EGFR* and *platelet-derived growth factor receptor (PDGFR) *are receptor tyrosine kinases (RTKs). In GBM, 40–60% of cases exhibit *EGFR* amplification and high EGFR protein expression levels [39, 40]. *PDGFR* is overexpressed at the transcriptional level. This *EGFR* activation initiates signal transduction such as the PI3K/Akt pathway. The phosphatidylinositol-3′ kinases (PI3Ks) are lipid kinases that are activated downstream of growth factor receptor signaling. Activated PI3Ks phosphorylate the lipid phosphatidylinositol (4, 5)-bisphosphate (PIP2) to generate phosphatidylinositol (3, 4, 5)-triphosphate (PIP3). PIP3 recruits Akt, which is the major effector of this pathway, to the cell membrane. Activated Akt enhances cell growth, survival, and proliferation and indirectly enhances the activity of mammalian target of rapamycin (mTOR), which controls cell growth by regulating various cellular processes. *PTEN* suppresses Akt phosphorylation by reversing PI3K-driven phosphorylation, resulting in the inhibition of the PIP3 signal thus suppressing cell proliferation [36, 41, 42]. Considering that these various alterations affect cell survival and proliferation, many studies suggest a novel strategy targeting these small molecules to improve the dismal prognosis of GBM [43].

To date, the small-molecule inhibitors of *EGFR *that were introduced in clinical trial include gefitinib, erlotinib, and nimotuzumab [44–46]. Gefitinib is an oral low-molecular-weight adenosine triphosphate mimetic of the anilinoquinazoline family. Gefitinib is an efficient therapeutic agent for a subset of patients with nonsmall-cell lung cancers (NSCLC), particularly the ones who harbor an activating *EGFR* mutation. However, several clinical trials evaluating the efficacy of gefitinib with or without TMZ in GBM report disappointing efficacy. Uhm et al. report that patients who exhibit adverse reactions to gefitinib (e.g., rash and diarrhea) have prolonged overall survival [47] Erlotinib is also an *EGFR* tyrosine kinase inhibitor (EGFR-TKI). This EGFR-TKI exhibits clinical activity particularly in tumors that have mutations in the adenosine triphosphate binding pocket of the tyrosine kinase domain of the *EGFR *gene. Although several clinical trials have evaluated the efficiency of erlotinib, almost all failed to demonstrate its efficiency or any additional benefit [45, 48]. Nimotuzumab was tested in an open-label randomized phase III trial in 150 patients with newly diagnosed GBM, but the interim analysis has failed to demonstrate efficacy [49]. *EGFR variant III (EGFRvIII)* is a constitutively activated mutation of *EGFR* that is expressed in approximately 25% of GBM cases but not in normal tissues. PF-04948568 is a vaccine containing a 13-amino acid sequence unique to EGFRvIII. A randomized phase IIb/III ACTIII study was initiated; the primary objective was to reject the null hypothesis that less than 53% of patients will be progression-free at 5.5 months from first vaccination [50]. Since this vaccination was well tolerated and the null hypothesis was rejected, further study is warranted. Afatinib, an irreversible erbB family blocker, exhibits high *in vitro* activity in tumor cell lines resistant to reversible EGFR inhibitors. This study compared the afatinib alone and afatinib with TMZ therapies with TMZ alone therapy [51]. The results show that afatinib alone is less effective than TMZ alone. Furthermore, afatinib with TMZ is comparable to TMZ alone.

Another TKI activating the PI3K/Akt/mTOR pathway is *PDGFR*. Imatinib blocks the ATP-binding site of tyrosine kinase proteins including *PDGFR* and inhibits the activity of *PDGFR*. The clinical efficiency of imatinib against other cancers such as chronic myeloid leukemia (CML) and gastrointestinal stromal tumors (GISTs) has been demonstrated [52–54]. In addition, since imatinib is active in GBM cell lines and mouse models, several clinical trials have evaluated its efficiency in GBM patients [43, 55, 56]. However, most of these clinical trials were not able to demonstrate any advantage of imatinib. It should be noted that most of these clinical trials to date enrolled unselected patients in whom the relative importance of each dysregulated molecule affecting tumor growth was largely unknown, which may be why these clinical trials demonstrate no advantage. Sunitinib is an orally available multitarget TKI of FDR, PDGFR, and c-kit. It exhibits broad-spectrum antiangiogenic activity. A phase II trial with stratification of prior use of BEV was designed for recurrent GBM to assess the safety and efficacy of 37.5 mg sunitinib administered on a continuous daily schedule. Twenty-eight and twenty-one patients have been enrolled in the BEV-resistant and BEV-naïve arms, respectively [57]. However, no patient has reached PFS6 at all. The efficacy of dasatinib, a PDGF and Src inhibitor, was evaluated retrospectively in recurrent malignant gliomas [58]. Twenty patients were treated with dasatinib alone, and in combinations with BEV and other anticancer drugs, or BEV-naïve. Dasatinib alone or in combination with BEV did not exhibit activity because low central nervous system penetration may limit its activity. 

### 3.3. Targeting the PI3K/Akt/mTOR Pathway

Following the activation of RTK, the activated PI3K/Akt/mTOR pathway induces cell growth and proliferation. In addition to the RTK inhibitors described above, several studies suggest potential therapeutic targets of PI3K, Akt, and mTOR [38, 59, 60]. A preclinical study demonstrated that LY294002 and wortmannin inhibit PI3K. Because of toxic effects, poor pharmaceutical properties, and a lack of selectivity, the use of these agents was restricted in the preclinical study. Recently, the thienopyrimidine drug GDC-0941 was found to exhibit excellent oral anticancer activity in a preclinical study and is now undergoing a phase I clinical trial in cancer patients [59]. In addition, imidazopyridines, pyridopyrimidines, quinazolyne derivatives, thiazoles, azolepyrimidine derivatives, and other chemotypes are reported to inhibit the PI3K. Akt is also heavily targeted for therapy. The phospholipid perifosine is suggested to interfere with the association of the Akt PH domain with PIP3, thus blocking the membrane localization of the protein. Perifosine is currently undergoing phase II clinical trials for prostate, head and neck, breast, and pancreatic cancers, melanomas, and sarcomas [61]. The mTOR kinase is intimately linked to PI3K/Akt signaling as well as the regulation of protein synthesis, cell growth, and survival. The dysregulation of mTOR is regarded as a therapeutic target. In addition, rapamycin and its analogs inhibit mTOR kinase via a rather indirect fashion. At present, 2 rapamycin analogs, temsirolimus and everolimus, are approved for the treatment of metastatic renal cell cancer [62]. Several clinical trials evaluating the efficiency of mTOR inhibitors such as temsirolimus in gliomas have been performed. The results of these trials suggest that monotherapy with temsirolimus does not prolong survival but combination therapy enhances its efficiency [43].

### 3.4. Glutamate Pathway

Glutamate is a major excitatory neurotransmitter in CNS. It is stored in synaptic vesicles and released as neurotransmitter. After it serves as neurotransmitter, it is rapidly took up at the plasma membrane of neurons, glial cells, and terminated. Glioma cells released glutamate in concentration, and glutamate reuptake is reduced due to reduction of glutamate transporter. This increased glutamate influences the surrounding cells and the glutamatergic system is associated with the proliferation, survival and migration of gliomas. Talampanel is an antagonist of the *α*-amino-3-hydroxy-5-methyl-4-isoxazolepropionic acid (AMPA) glutamate receptor. The phase 2 trial of Talampanel combined with conventional TMZ and radiation for 72 newly diagnosed GBM patients showed median survival time of 18.3 months. This trial suggested the efficiency of Talampanel [63]. The other phase 2 trial evaluated the efficiency of Talampanel as single agent for recurrent malignant glioma patients. This trial shows no significant activity as single agent (median OS; 13 weeks) [64]. 

### 3.5. Histone Deacetylase

Vorinostat is an oral histone deacetylase (HDAC) inhibitor. HDAC acts on nucleosomal histones, leading to the tight coiling of chromatin and silencing of the expression of various genes. HDAC regulates cell survival, proliferation, tumor cell differentiation, cell cycle arrest, and apoptosis. There is preclinical evidence that vorinostat has antitumor activity against malignant glioma cell lines *in vitro* and orthotopic xenografts *in vivo*. Animal experiments also support the conclusion that vorinostat crosses the blood-brain barrier [65]. On the basis of these results, a phase I study of vorinostat in combination with TMZ was performed and revealed that this treatment is well tolerated; a phase II trial is under way [66].

## 4. Strategy Targeting Glioma-Initiating Cells

Several studies revealed that gliomas harbor a small population of cells termed glioma stem-like cells (GSCs) [67, 68]. GSCs have the ability to undergo self-renewal and initiate tumorigenesis. GBM forms extensively heterogeneous bulk tumors; this heterogeneity may be crucial for treating this disease. The presence of GSCs may be an important clue in clarifying the details of this heterogeneity. In addition, GSCs are resistant to a wide variety of chemotherapeutic agents and possess a remarkable ability to recover from cytotoxic therapy [69]. Furthermore, GSCs play a crucial role in RT failure, as tumors surviving RT are enriched in GSCs. Therefore, an alternative strategy involving selective targeting of this functionally distinct chemo- and radiation-resistant small group of GSCs rather than the bulk of the tumor may be more successful in treating this deadly disease [70]. GSCs exhibit various alterations to signaling pathway activity including PTEN, sonic hedgehog (SHH), notch, wingless-type MMTV integration site family member (WNT), maternal embryonic leucine-zipper kinase (MELK), and B lymphoma Mo-MLV insertion region 1 (BMI1), which are associated with self-renewal and neoplastic proliferation. These altered pathways may represent possible targets for GSCs.

### 4.1. PI3K/Akt/mTOR Pathway Including the PTEN Pathway

PTEN suppresses Akt phosphorylation by reversing PI3K-driven phosphorylation, resulting in the inhibition of PIP3 signaling and the suppression of cell proliferation. The *PTEN* gene is mutated in 15–40% of primary GBM cases. PTEN deletions with retinoblastoma-associated protein (pRb) inactivation or ABCG2 transporter activation accelerate the formation of aggressive high-grade tumors and GSC-like neurosphere formation capacity in a transgenic mouse model of glioma [71–75]. Although PTEN is one of the most remarkable targets involved in GSC activity, its status in GSCs has yet to be elucidated. The dysfunction of PTEN leads to the activation of the PI3K/Akt/mTOR pathway. Therefore, therapy targeting the PI3K/Akt/mTOR pathway described above may be also effective against GSCs exhibiting PTEN dysfunction. Thus, these results suggest the potential of efficiency of such therapies for GSCs.

### 4.2. SHH Pathway

SHH is a secreted protein critical for pattern formation in the central nervous system. SHH ligand binding to its receptors, patched homolog (PTCH) and smoothened homolog (SMO), leads to the activation of gliotactin (GLI) transcription factors that are translocated into the nuclease to regulate various cellular activities, including the maintenance of cell stemness, survival, and proliferation. Altaba et al. demonstrate that SHH signaling regulates the expression of stemness genes such as *Nanog homeobox* (*NANOG*), *SRY-box containing gene 2* (*SOX2*), and *octamer-binding protein 4* (*OCT4*). In addition, they demonstrate that SHH-GLI signaling is required not only for sustained glioma growth and survival but also for GSC survival and proliferation [76]. Considering these reports, the inhibition of the SHH signaling pathway may be a target of therapy. The novel SMO inhibitor, vismodegib (GDC-0449), exhibits antitumor activity in a mouse model of medulloblastoma and primary human tumor cell xenograft models including colorectal cancer and pancreatic carcinoma. A phase I clinical trial was initiated on the basis of these preclinical tests; the results demonstrate that vismodegib is generally well tolerated with an acceptable safety profile in refractory locally advanced metastatic solid tumors, including basal cell carcinoma and medulloblastomas [77]. Therefore, the SHH signaling pathway may be a potential target for therapy against GSCs.

### 4.3. Notch Pathway

The Notch pathway is initiated by the binding of transmembrane ligands on one cell to the notch receptors on an adjacent cell. This binding causes the **γ**-secretase-mediated proteolytic release of the Notch intracellular domain (NICD). Released NICD translocates into the nucleus and then turns CSL (a transcriptional factor) from a repressor to an activator, causing various effects [78]. Notch controls the specification, proliferation, and survival of nonneoplastic neural precursors and is aberrantly activated in embryonal brain tumors, suggesting a molecular link between neural stem cells and medulloblastomas. Previously, Sullenger et al. demonstrated that GSCs promote radioresistance compared with GBM tumor bulk because GSCs preferentially activate the DNA damage-response pathway. Notch signaling plays an important role in this DNA damage response pathway via the activation of the PI3K/Akt pathway and the pro-survival protein, Mcl-1. Notch pathway inhibition using *γ*-secretase inhibitors (GSIs; MK0752) impairs cell growth, clonogenic survival, and tumor formation ability and sensitizes GSCs to radiation at clinically relevant doses [79, 80].

### 4.4. Wingless-Type MMTV Integration Site Family Member (Wnt) Pathway

Wnt signals are divided into 2 different pathways: the canonical, or WNT/*β*-catenin pathway is involved in cell fate determination and the noncanonical pathway is involved in the control of cell movement and tissue polarity [35]. Following the binding of Wnt protein to a receptor complex comprising Frizzleds/low-density lipoprotein receptor-related protein (Fz/LRP), cytoplasmic disheveled (Dvl) is phosphorylated. The phosphorylation of Dvl inhibits the activity of glycogen synthase kinase-3*β* (GSK-3*β*), elevating nonphosphorylated *β*-catenin levels in the cytoplasm. *β*-Catenin translocates into the nucleus and forms a complex with members of the T-cell transcription factor (TCF)/lymphoid enhancer-binding factor (LEF) family of transcription factors [81]. Epigenetic silencing and loss-of-function mutations of negative regulators of WNT signaling are observed in a variety of human cancers. It is suggested that Wnt signaling is also involved in the regulation of cancer stem cells because of the many similarities in the pathways that regulate normal and cancer stem cells. Therefore, the inhibition of Wnt signaling may disrupt the maintenance of the stemness of GSCs. Although they include preclinical agents, several agents inhibiting the Wnt pathway are suggested for potential clinical use in a review by Takahashi-Yanaga and Kahn [82]. Of these agents, those that are used clinically are NSAIDs such as aspirin, sulindac, and celecoxib; celecoxib is the only NSAID approved by the Food and Drug Administration (FDA) for the treatment of familial adenomatous polyposis. The inhibition of the Wnt pathway by celecoxib has been shown by its ability to induce the degradation of TCF. 

These various pathways altered in GBM or in the presence of GSCs with altered signaling pathways may induce resistance to conventional therapy. In addition, in other cancers, several studies suggest the efficiency of various small-molecule inhibitors. Although several clinical trials of these inhibitors in GBM have been performed, almost all failed to demonstrate the efficiency of these inhibitors compared with conventional therapy. We expect that combinations of these agents may overcome resistance to treatment or change the definition of patients who should be treated by each agent to induce a more favorable response.

## 5. Bypassing the Blood-Brain Barrier

The blood-brain barrier blocks most molecules that are larger than ~500 Da. Many drugs are denied access to the very regions where they would be effective, thus limiting the clinical application of most anticancer drugs for treating brain tumors. Each anticancer agent showed various permeability for BBB, although the relationship of its permeability to therapeutic efficacy is not clear [83]. Although several local therapies are attempted to overcome this BBB or “blood-tumor barrier,” local therapies should be more developed to deliver therapeutic agents in more distant locations due to highly infiltrative nature of high-grade gliomas.

### 5.1. Gliadel (Carmustine, BCNU) Wafers

A meta-analysis combining the results of the randomized phase III trial published by Westphal et al. [84] and an earlier randomized phase III study by Valtonen et al. [85] demonstrates that the subgroup of GBM treatments with BCNU wafers increases mean survival to 13.1 months compared with 10.9 months for placebo patients (*P* = 0.03). The results of the 2 trials led the FDA to approve Gliadel for the treatment of newly diagnosed GBM in 2003. A combination of local BCNU wafer treatment and concomitant radiochemotherapy with TMZ is attractive not only because it may significantly reduce the toxicity of a systemic combination of BCNU and TMZ as described above but also because it may take advantage of the sensitizing effect of TMZ and BCNU on their respective resistance by MGMT [86]. However, several complications are associated with the implantation of BCNU wafers, including cerebral edema, healing abnormalities, cerebral spinal fluid leaks, intracranial infections, seizures, hydrocephalus, and cyst formation. The rates for these adverse events were well established in 2 randomized phase III trials that compared BCNU wafers with placebo ones. Gliadel wafer implantation may add to the toxicity of concomitant radiochemotherapy with systemic TMZ. Therefore, the combined approach requires special attention [87].

### 5.2. Convection-Enhanced Delivery

A direct intracerebral approach called convection-enhanced delivery (CED) may be used as a strategy to address these issues [88–90]. CED employs positive pressure that generates a local pressure gradient to distribute agents in the extracellular space. Unlike diffusion delivery, CED is not significantly affected by the concentration, molecular weight, or particle size of the agent. Furthermore, CED ensures high concentrations and the homogenous distribution of a drug throughout a given target tissue.

### 5.3. CED of IL13-PE38QQR (Cintredekin Besudotox) for Recurrent GBM: The PRECISE Study

Interleukin (IL)-13 is a cytokine derived from type 2 T-helper cells and can bind to 2 receptor chains: IL-13R*α*1 and IL-13R*α*2; IL-13 has low affinity for the IL-13R*α*1 chain and high affinity for the IL-4R*α* chain. It forms a receptor complex with the IL-4R*α* chain, which is involved in IL-13-induced signal transduction via either Janus kinase/signal transducer and activator of transcription (JAK-STAT) or PI3K [91]. The IL-13R*α*2 chain binds to IL-13 with high affinity and internalizes it after ligand binding without the involvement of other chains.

IL-13R is overexpressed in a majority of glioma cell lines and resected GBM specimens [92]. A chimeric fusion protein composed of human IL-13 and mutated forms of *Pseudomonas aeruginosa* exotoxin A (PE38QQR) has been developed and shown to affect the specific cytotoxicity of glioma cell lines [92, 93].

IL-13-PE is reported to be more active against glioma cell lines than IL-4-targeted toxins *in vitro* [93]. In a phase I trial, 51 patients with GBM were administered IL-13-PE38QQR via CED [94]. A phase III study was conducted to compare the efficacy of IL-13-PE to that of Gliadel wafers in patients with malignant gliomas [93]. PFS was longer (17.7 versus 11.4 weeks) in patients treated with IL-13-PE than in patients treated with Gliadel wafers; however, there was no significant difference in the median survival time between the 2 groups.

Overall, IL-13-based toxins can be potentially used in adjuvant therapy for malignant gliomas, but their use requires further clinical studies.

### 5.4. CED of TGF-*β* Antisense: The SAPPHIRE Study

Transforming growth factor-*beta* (TGF-*β*) is a multifunctional regulatory polypeptide belonging to a ligand superfamily that includes the TGF-*β*s, activins, and bone morphogenetic proteins (BMPs). TGF-*β* controls many aspects of cellular function including proliferation, differentiation, migration, apoptosis, adhesion, angiogenesis, immune surveillance, and survival. Three TGF-*β* isoforms have been found: TGF-*β*1, TGF-*β*2, and TGF-*β*3. The TGF-*β*2 isoform is specifically overexpressed in malignant gliomas. Increased TGF-*β*2 levels are associated with advanced disease stage and cause immunodeficiencies in patients with gliomas [95]. TGF-*β*2 not only inhibits lymphocyte proliferation but also has multiple effects on the immune system. These effects include inhibition of immune cell activation, blockage of antitumor activity, shift in cytokine balance toward immunosuppression, and inhibition of antigen presentation. Thus, the targeted inhibition of TGF-*β*2 should have an antitumor effect and allow an immune-mediated response. Several approaches to block TGF-*β* function are currently being studied including the use of monoclonal antibodies against TGF-*β*, recombinant fusion proteins containing the ectodomains of TGF-*β* receptor (T*β*R)II and T*β*RIII to prevent the binding of TGF-*β* ligands, ATP competitive inhibitors at the ATP-binding site of T*β*RI kinase, and antisense oligonucleotides specific for TGF-*β*2 [96–101].

Trabedersen (AP-12009) is a synthetic antisense oligodeoxynucleotide designed to block TGF-*β*2 production [102]. In a randomized controlled phase IIb trial involving patients with brain tumors, the survival rates of patients for whom trabedersen was intratumorally administered were higher than those of patients receiving standard chemotherapy [103]. An international clinical phase III trial is currently recruiting patients with recurrent or refractory anaplastic astrocytoma. A randomized controlled dose-finding phase IIb study evaluated the efficacy and safety in 145 patients with recurrent or refractory high-grade gliomas [104]. The patients were randomly assigned to receive 10 or 80 *μ*M trabedersen or standard chemotherapy. The primary end point was 6-month tumor control rate. Although this study failed to meet the primary end point, it could be due to the pseudoprogression that occurs with immune therapies. A prescribed anaplastic astrocytoma subgroup analysis found a significant benefit for 10 *μ*M trabedersen with respect to the 14-month tumor control rate. The 2-year survival rate for 10 *μ*M trabedersen tended to be superior to those of the other treatments. An international clinical phase III trial is currently recruiting patients with recurrent or refractory anaplastic astrocytomas with end points of 14-month progression rate and 2-year survival rate.

## 6. Future Perspectives

The Cancer Genome Atlas (TCGA) is a project that catalogs genomic abnormalities that are involved in the development of cancer [105, 106]. TCGA published the results of its first study in a large GBM cohort consisting of 206 patient samples. Techniques that are currently used for detection of abnormalities include gene-expression profiling, copy-number variation profiling, single-nucleotide polymorphism (SNP) genotyping, genome-wide methylation profiling [107], microRNA profiling [108], and exon sequencing. Since the publication of the first paper, several analysis groups within the TCGA Network have presented the results of highly detailed analyses of GBM. Verhaak et al. [109] recently subclassified GBM into proneural, neural, classical, and mesenchymal subtypes by integrating multi-dimensional data on gene expression, somatic mutations, and DNA copy number. The major features of the Proneural class are focal amplification of *PDFRA*, *IDH1* mutation, and *TP53 *mutation and/or loss of heterozygosity. Moreover, the group showed high expression of genes associated with oligodendrocyte development, such as *PDGFRA, NKX2-2*, and *OLIG2*. The Neural subtype is characterized by the expression of neuron markers such as *NEFL*, *GABRA1*, *SYT1*, and *SLC12A5*. The Classical subtype features high *EGFR* expression associated with chromosome 7 amplification and low expression of *p16INK4A* and *p14ARF*, resulting from focal 9p21.3 homozygous deletion. Neural stem cell markers such as nestin, as well as components of the Notch and Sonic hedgehog signaling pathways, are highly expressed in the Classical type. The Mesenchymal subtype is characterized by focal hemizygous deletions at 17q11.2 that contains *NF1* and high expression of *YKL-40 (CHI3L1)*, *MET*, *CD44*, and *MERTK*. Genes in the tumor necrosis factor superfamily pathway and NF-kappaB pathway, such as *TRADD*, *RELB*, and *TNFRSF1A*, are highly expressed in this subtype. The classification of GBM may lead to establishment of personalized therapies for groups of patients with GBM. However, the results of clinical studies of EGFR and PDGFR inhibitors as monotherapy are disappointing thus far. While research and development of more promising molecular-targeted agents are needed in the laboratory, molecular-targeted agents are likely to have synergistic antitumor effects in combination. On the other hand, strategies of how to evaluate better ways to design early phase clinical trials, to choose better endopoints should avoid trials that will not provide helpful answers. The discrepancy between PFS and OS as endpoints are still controversial; also the question when and how to integrate new therapies into the backbone of standard therapy still remains.

## Figures and Tables

**Figure 4:**
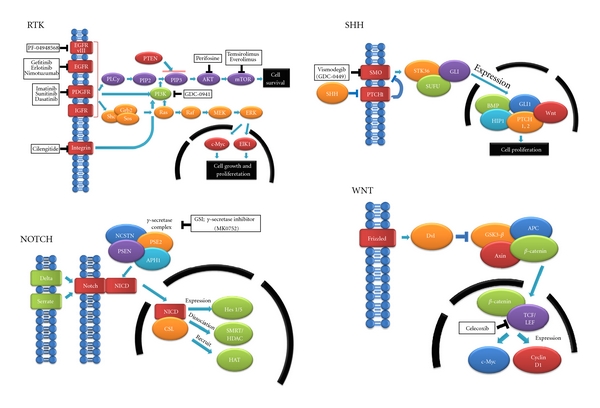
Altered signaling pathways and inhibitors. Receptor tyrosine kinase (RTK), *EGFR* and *platelet-derived growth factor receptor (PDGFR) *are receptor tyrosine kinases (RTKs). In GBM, 40–60% of cases exhibit *EGFR* amplification and high EGFR protein expression levels. *PDGFR* is overexpressed at the transcriptional level. *EGFR* activation initiates signal transduction such as the PI3K/Akt pathway. *EGFR variant III (EGFRvIII)* is a constitutively activated mutation of *EGFR*, that is, expressed in approximately 25% of GBM cases but not in normal tissues; Sonic Hedgehog (SHH), SHH is a secreted protein critical for pattern formation in the central nervous system. SHH ligand binding to its receptors, patched homolog (PTCH) and smoothened homolog (SMO), leads to the activation of gliotactin (GLI) transcription factors that are translocated into the nuclease to regulate various cellular activities, including the maintenance of cell stemness, survival, and proliferation; NOTCH, The Notch pathway is initiated by the binding of transmembrane ligands on one cell to the notch receptors on an adjacent cell. This binding causes the **γ**-secretase-mediated proteolytic release of the Notch intracellular domain (NICD). Released NICD translocates into the nucleus and then turns CSL (a transcriptional factor) from a repressor to an activator, causing various effects; WNT, Wnt signals are divided into 2 different pathways: the canonical or WNT/*β*-catenin pathway is involved in cell fate determination and the noncanonical pathway is involved in the control of cell movement and tissue polarity [35]. Following the binding of Wnt protein to a receptor complex comprising Frizzleds/low-density lipoprotein receptor-related protein (Fz/LRP), cytoplasmic disheveled (Dvl) is phosphorylated. The phosphorylation of Dvl inhibits the activity of glycogen synthase kinase-3*β* (GSK-3*β*), elevating nonphosphorylated *β*-catenin levels in the cytoplasm. *β*-Catenin translocates into the nucleus and forms a complex with members of the T-cell transcription factor (TCF)/lymphoid enhancer-binding factor (LEF) family of transcription factors.
